# Analysis of the decomposition of an anhydride-cured epoxy resin by subcritical hydrolysis

**DOI:** 10.1038/s41598-025-13375-8

**Published:** 2025-07-29

**Authors:** Simon Backens, Arthur Konrad Wieland, Stefan Schmidt, Wilko Flügge, Lukas Friederici, Christopher Rüger

**Affiliations:** 1 Fraunhofer Institute for Large Structures in Production Engineering IGP, 18059 Rostock, Germany; 2 Joint Mass Spectrometry Centre/Chair of Analytical Chemistry, University of Rostock, 18059 Rostock, Germany; 3 Department Life, Light & Matter (LLM), University of Rostock, 18059 Rostock, Germany

**Keywords:** Subcritical hydrolysis, Anhydride-cured epoxy resin, Glass transition temperature, Core-shrinking model, Gas chromatography, Mass spectrometry, Composites, Mass spectrometry

## Abstract

The decomposition of an anhydride-cured epoxy resin by subcritical hydrolysis is studied under variation of reaction temperature, decomposition duration and water volume using a batch reactor. Within the framework of a design of experiment, the process is evaluated by the gravimetric decomposition of the epoxy resin clearly showing that temperature is the most important factor. Duration and water volume have only smaller positive effects. The mass loss mechanism of the cube-shaped specimens corresponds to a heterogeneous surface degradation where material is only lost from the outer areas. The determination of glass transition temperatures after the experiments demonstrates that the water hardly affects the center of the epoxy cubes. Therefore, a core-shrinking model is successfully applied for analysis of the decomposition kinetics. The product distribution in the aqueous phase reveals a large number of organic compounds. Higher temperatures, longer durations and lower water volumes lead to an increase in decomposition product complexity and concentrations, but the degradation pathways are apparently not affected.

**Supplementary Information:**

The online version contains supplementary material available at 10.1038/s41598-025-13375-8.

## Introduction

Due to their good mechanical properties and high corrosion resistance, epoxy resins (EP) are the predominant matrix system for carbon fiber reinforced plastics (CFRP) in aircrafts and mechanically highly stressed components of wind turbines^1,2^. Large quantities of end-of-life CFRP are expected to come primarily from the commercial aeronautical and wind power sector in the next few years^3,4^. For these reasons, efforts to recycle CFRP are largely focused on epoxy-based composites.

Chemical recycling of CFRP by deliberate decomposition of the epoxy resin can be performed with a variety of different media under different process conditions^5–8^. In recent years, the use of sub- and supercritical water has received increased attention. There have been recycling studies with CFRP based on amine-^9–14^ and anhydride-cured EP^15–17^. Furthermore, the decomposition of pure amine-^18–20^ and anhydride-based EP^16,20–22^ has been investigated.

**Fig. 1:**
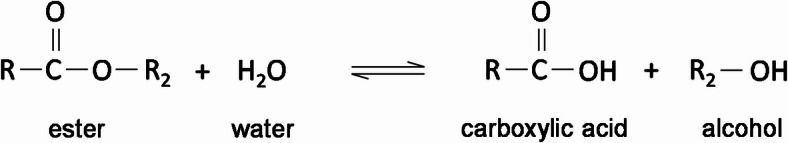
Reaction of ester hydrolysis, adapted from^24^.

Due to the ester groups in the polymer structure, anhydride-cured EP (ANH-EP) has a particular susceptibility to water^23–25^. The chemical reaction, called ester hydrolysis (Fig. 1), produces carboxylic acid and alcohol and can be catalyzed by both bases and acids^26^.

Gong et al.^20^ confirmed that the subcritical hydrolytic decomposition of anhydride-cured diglycidyl ether of bisphenol A (DGEBA) proceeds more easily than the decomposition of amine-cured DGEBA on account of the ester groups. While the EP system with anhydride hardener could be completely degraded at 275 °C without catalyst in 60 min, the system with amine hardener required 60 min at 330 °C.

Liu Yuyan et al.^21^ also decomposed ANH-EP under subcritical conditions without any catalyst. When analyzing the effect of pressure on the reaction, they concluded that too much water inside the reactor remaining in the liquid state has a negative influence on the reaction. They argued that the liquid water covers the surface of the specimens imposing interphase mass transfer restrictions. Using differential scanning calorimetry, they showed that the glass transition temperature of the solid products after hydrolytic treatment decreased with increasing reaction time at 230 °C due to decreasing crosslinking.

Okajima et al.^16^ studied the influence of different catalysts on the hydrolytic decomposition of ANH-EP at temperatures between 300 and 400 °C and reaction times up to 240 min. They found the best results when using potassium carbonate. But the use of sodium carbonate as well as potassium and sodium hydroxide also increased the yield of phenolic monomers compared to no catalyst.

Liu Yuyan et al.^22^ studied the effect of sulfuric acid (H_2_SO_4_) and potassium hydroxide (KOH) on the chemical decomposition of ANH-EP in nearcritical water at 270 °C with a reaction time of 45 min. They determined optimal concentrations of 0.4 mol/L for H_2_SO_4_ and 0.5 to 1.0 mol/L for KOH. The authors ascribed the positive influence of the catalysts to increased concentrations of hydronium and hydroxyl ions, respectively.

However, information on mass loss mechanisms of ANH-EP at subcritical conditions is not reported. This may be mainly due to the small sample masses (0.2 g^16^, 1 g^20,22^) that were decomposed in the studies.

The mass loss mechanisms of ANH-EP differ depending on the environmental conditions. At low hydrolysis rates homogeneous bulk degradation dominates, at high hydrolysis rates heterogeneous surface degradation prevails^27^. Bulk degradation (or erosion) means that material is lost from the entire volume of the polymer, surface degradation that it is progressively lost only from the specimen surface^28^. If water diffuses into the polymer faster than polymer bonds degrade, bulk erosion takes place. If, however, polymer bonds degrade faster than water diffusion proceeds, surface erosion occurs^29^.

The relationship between resin decomposition and reaction time has been previously studied by application of either Arrhenius-type Eqs^9,30–32^ or reaction rate limited shrinking core model (SCM)^31–34^. Only Pinero et al.^9^ used water as solvent, but they recycled CFRP based on amine-cured EP. So, there are no results for ANH-EP yet.

Hydrolysis has a strong influence on the glass transition temperature (T_g_) of polymers. On the one hand, it is known that T_g_ of an epoxy resin is directly dependent on the degree of crosslinking^35^ and that the crosslink density can be studied, among other methods, via T_g_ shifts^36^. Therefore, a decrease in T_g_ can be considered as an indicator of the degradation of crosslinks. On the other hand, the uptake of low molecular weight substances such as water leads to plasticization and also contributes to a T_g_ decrease^37^. ANH-EP, though, are only moderately hydrophilic. Swelling and plasticization therefore only play a minor role^38^.

In this work, the decomposition of an anhydride-cured epoxy resin by subcritical hydrolysis is studied under variation of reaction temperature, decomposition duration and water volume within a design of experiments (DoE). The DoE is evaluated by the gravimetric decomposition of the epoxy resin specimens. The decomposition characteristics are described to illustrate the mass loss mechanism of the ANH-EP. Selected specimens are examined by differential scanning calorimetry (DSC) in order to detect differences and changes in glass transition temperatures caused by the hydrolysis process. Based on these results, the decomposition kinetics are analyzed. The effect of different process conditions on the product distribution in the aqueous phase is studied by gas chromatography separation with mass spectrometric detection (GC-MS).

## Materials and methods

### Preparation of epoxy resin specimens

LITESTONE^®^ epoxy resin 3100E, a bisphenol A diglycidyl ether, and LITESTONE^®^ hardener 3102 H, a mixture from tetrahydromethylphthalic anhydride (> 80%), polypropylene glycol (10–20%) and benzyltriethylammonium chloride (1–5%), were used to produce three epoxy resin blocks with dimensions of 120 mm x 100 mm x ca. 30 mm. For this purpose, resin and hardener were homogeneously mixed under a vacuum. Subsequently the mixture was filled free of voids in three silicone molds. The first step of curing was carried out for 72 h at 30 °C in a climate cabinet in order to slowly gel and to avoid too much heat development caused by the exothermic reaction. The process was then continued for 1 h each at 100, 150 and 210 °C in a convection oven. Finally, identical cubic specimens with an edge length of 25 mm and a mass of about 18.6 g were prepared from the three blocks using a CNC milling machine.

### Decomposition of epoxy resin specimens

The decomposition experiments were carried out in a high pressure, high temperature, stainless steel reactor (novoclave, Büchi AG). The laboratory batch-type reactor could be electrically heated and cooled with water. The specimens were placed in a stainless steel mesh basket in order to prevent direct contact with the reactor bottom or wall. The basket was located at the bottom of the reactor so that the epoxy cubes were completely immersed in the water.

A design of experiments (DoE) was set up based on three controlled parameters and a target value. Reaction temperature, decomposition duration at this temperature and volume of deionized water were chosen as factors and varied on three levels (Table 1) on the basis of preliminary tests, resulting in a total of 18 experiments. The gravimetrically determined degree of decomposition of the specimens was defined as target value. The statistical analysis of the DoE results was performed using the software Visual-XSel 16.0.

**Table 1:** Factors and levels of the DoE.

Factor	Unit	Level 1	Level 2	Level 3
Reaction temperature	°C	250	275	300
Decomposition duration	min	15	30	45
Water volume	ml	100	200	300

Prior to the decomposition experiments, the epoxy resin specimens were conditioned for at least 24 h in a desiccator located in a laboratory at standard climate (23 °C and 50% RH) and subsequently weighed. Then, they were put in the mesh basket inside the reactor and deionized water was added. The reactor was sealed and heated at 5 °C/min to the reaction temperature. This heating rate was kept constant for all experiments. The reaction temperature was maintained for the decomposition duration. The reactor was then water-cooled using the built-in cooling pipes. The cooling was always performed as quickly as possible, which took about 35 to 40 min to reach 100 °C, and 65 to 75 min to get the temperature down to 50 °C.

Finally, the reactor was opened and the mesh basket with the epoxy specimen – if still existent – was removed. After drying in a desiccator to mass consistency for 24 h, the specimens were weighed again. In addition, the edge lengths of the cubes were measured in all three spatial directions as x, y and z with a digital caliper for analysis of the decomposition kinetics. The liquid aqueous phase was collected in glass bottles.

### Analysis of epoxy specimens

The degree of decomposition D was determined using Eq. (1)

1 $$\:D = \:\left[ {\left( {m_{0} - m_{1} } \right)/m_{0} } \right]\: \cdot 100\:\%$$

where m_0_ represents the specimen mass before the experiment and m_1_ the mass of the dried solid specimen after the subcritical hydrolysis.

The glass transition temperatures of the three cast and cured epoxy resin blocks were determined by differential scanning calorimetry (DSC) according to DIN EN ISO 11357-2. DSC was performed using a DSC 1 (Mettler Toledo). The samples were heated from 20 °C to 200 °C at a heating rate of 10 K/min under nitrogen atmosphere. Three measurements were carried out for each of the resin blocks and averaged.

Selected specimens were also analyzed by DSC after subcritical hydrolysis. For this purpose, samples were prepared from the edge and the center of the cubic epoxy specimens. Two measurements were carried out in each case, the results were averaged.

### Analysis of decomposition kinetics

The core-shrinking model (CSM) was applied for the analysis of the reaction kinetics. The decomposition of epoxy resin can therefore be expressed as^39^:

2 $$\:X=1-\:\frac{V}{{V}_{0}}=1-\:\frac{xyz}{{L}^{3}}$$

where V, in mm³, is the volume of the epoxy specimen at a specific time of the reaction, calculated via the three edge lengths x, y and z, in mm, at this specific time and V_0_ is the specimen volume, in mm³, before decomposition calculated via the initial edge lengths L in mm.

In case of shape similarity, it can be assumed that^39^:

3 $$\:\frac{x}{L}=\:\frac{y}{L}\:=\:\frac{z}{L}=M$$

Substituting Eq. (3) into Eq. (2) gives

4 $$\:X=1-\:\frac{{L}^{3}{M}^{3}}{{L}^{3}}=\:1-\:{M}^{3}$$

resulting in:

5 $$\:M=\:{\left(1-X\right)\:}^{1/3}$$

Inclusion of an induction period t_I_ finally gives^39^:

6 $$\:1-\:{\left(1-X\right)\:}^{1/3}=1-M\:=\:{k}_{s}{\prime\:}\:(t-\:{t}_{I})$$

where k_s_’, in min^−1^, is the apparent rate constant of the epoxy resin and t_I_, in min, is the induction period. While the rate constant can be determined from the slope of the line of best fit, the induction period can be determined from the intercept with the x-axis.

### Analysis of the liquid aqueous phase

The liquid aqueous phase was analyzed for the amount and kind of decomposition products from the epoxy resin. The aqueous phase was extracted with dichloromethane (DCM) to determine the organic composition of dissolved degradation products in water. Therefore, 2 ml of sample were extracted thrice with 1 ml DCM, acidified and stored in a freezer at − 20 °C upon analysis. The extracts from the 300 °C experiments were diluted in a ratio of 1:10 before the GC-MS measurement. The extracts from the 250 and 275 °C experiments were not diluted. A 7820 A GC (Agilent Technologies Inc.) with a Split/Split less injector (1 μm volume) and a split ratio of 1:10 was used. The gas chromatograph was equipped with a 30 m DB-5 capillary column (250 μm inner diameter, 0.25 μm film, helium at 99.999%). A constant flow rate of 1.5 ml/min was used for the following temperature program: hold for 4 min at 50 °C, ramp to 320 °C with 10 K/min, and hold for 5 min. A solvent delay of 4 min was used. Afterwards the effluent from the column was ionized with 70 eV and analyzed by a 5977E MSD (Agilent Technologies Inc.). The obtained data were analyzed with the software package AMDIS to identify features in the chromatograms and assigned to the NIST spectral library. Furthermore, the mass spectra and chromatograms were processed with a self-written MATLAB (R2023a) routine to visualize the results.

## Results and discussion

### Design of experiments

The parameter combinations, the corresponding decomposition, the weight percentages of phenol in the liquid fraction and the relative intensity of 1,2-propanediol, 3-phenoxy- (PDP) as compound with the highest abundance of the 18 experiments are shown in Table 2.

**Table 2:** Conditions used in the DoE with the results of the hydrolytic process defined by decomposition, phenol in the liquid fraction and relative intensity of 1,2-propanediol, 3-phenoxy- (PDP) as compound with the highest abundance.

No.	Temperature [°C]	Duration [min]	Volume [ml]	Decomposition [%]	w(phenol) [g/l]	Rel. Intensity PDP [%]
DOE 15	300	45	300	100	5.77	53.6
DOE 16	300	45	200	100	7.64	52.5
DOE 11	300	45	100	100	6.89	43.1
DOE 8	300	30	300	100	4.34	57.1
DOE 6	300	15	300	77.8	0.75	58.7
DOE 7	300	15	100	75.2	1.71	37.6
DOE 9	275	45	300	93.1	1.19	34.8
DOE 1	275	30	200	74.9	0.68	36.4
DOE 17	275	30	200	63.4	0.38	18.8
DOE 18	275	30	200	75.3	0.76	24.8
DOE 12	275	30	100	53.3	1.59	25.8
DOE 4	275	15	200	35.3	0.19	5.2
DOE 5	250	45	300	40.9	0.18	1.9
DOE 13	250	45	100	21.7	0.19	1.2
DOE 3	250	30	200	2.3	0.17	0
DOE 2	250	15	300	−2.5	0	0
DOE 10	250	15	100	−2.6	0	0
DOE 14	250	15	100	−2.3	0	0

**Fig. 2:**
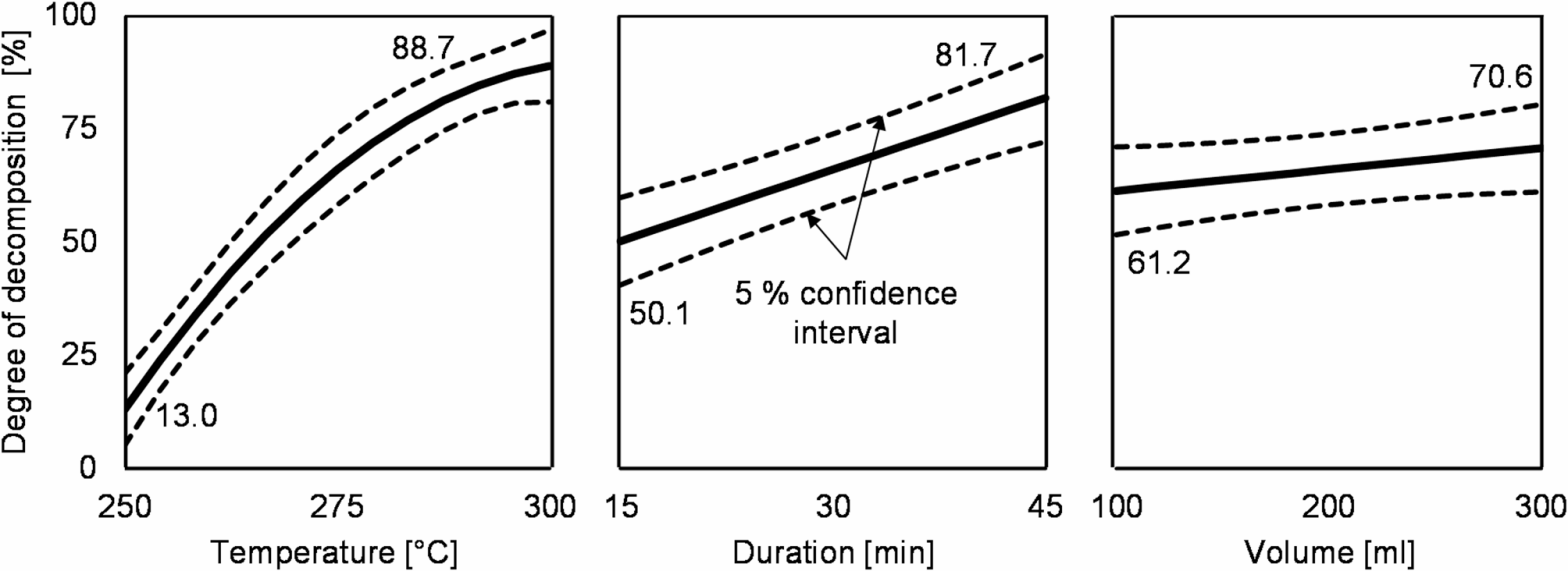
Influence and effects of temperature, duration and volume (of water) on the degree of decomposition.

The statistical evaluation of the DoE results is illustrated in Fig. 2 showing the influence of all three factors on the degree of decomposition under the assumption that one factor is changed at a time, while the others remain at medium level. The effect of a factor is defined as the change of the target value within the setting limits. The dotted lines represent the 5% confidence interval.

The reaction temperature has the biggest effect of about 75% points (pp) when raised from 250 to 300 °C. The properties of the water that change with increasing temperature (such as density and viscosity^40^) have a very favorable influence on the decomposition of the epoxy resin. The decomposition duration has a smaller but still significant effect of 31.6 pp. At all temperature levels, a significant increase in the degree of decomposition can be achieved with increasing duration. The water volume, however, has an effect of only 9.4 pp. This small increase in the target value is within the confidence interval. The parameter is therefore not significant according to the statistical evaluation.

These findings are in accordance with Pinero-Hernanz et al.^9^ and Oliveux et al.^18^, who studied the hydrolysis of CFRP and pure epoxy resin, respectively, by means of DoE – albeit amine-cured. They also found temperature to be by far the most important factor regardless of whether its increase was accompanied by a transition to the supercritical region^9^ or not^18^. An increase in time and water volume (or a decrease in the ratio of matrix to water) also had a positive effect. Oliveux et al. attributed the latter to a possible saturation of the solvent with decomposition products of the matrix.

### Decomposition characteristics

**Fig. 3:**
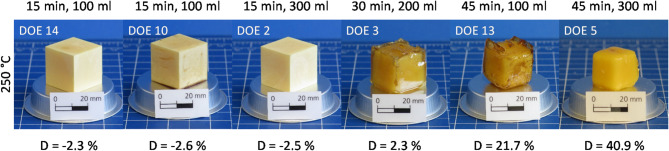
Epoxy specimens after hydrolysis at 250 °C with different decomposition durations and water volumes.

At a temperature of 250 °C (Fig. 3), the epoxy specimens become heavier within 15 min due to water absorption. Since ANH-EP has a relatively low hydrophilicity^38^, the increase in mass is only about 2.5%. Significantly higher water absorption would be expected for amine-crosslinked EP^38^. This mass increase is accompanied by a similar increase in volume (close to 3%). After 30 min, the first gravimetric decomposition can be determined, as shown by the small mass loss of 2.3%. A 45 min treatment already leads to a decomposition of up to 40.9%. Therefore, it can be expected that a further extension of duration would lead to complete decomposition of the epoxy cubes.

The appearance of the yellow specimens also changes with decomposition duration. Water absorption comes along with a pale color, the cube surfaces appear slightly porous (DOE 14, 10 and 2). With beginning decomposition, the surfaces of the cubes become transparent before dissolving (DOE 3 and 13). Finally, the transparent edge areas disappear, and a smaller, strong yellow epoxy cube remains (DOE 5). The mass loss mechanism evidently corresponds to the heterogeneous surface degradation. The polymer bonds apparently degrade faster than water can diffuse into the specimens.

**Fig. 4:**
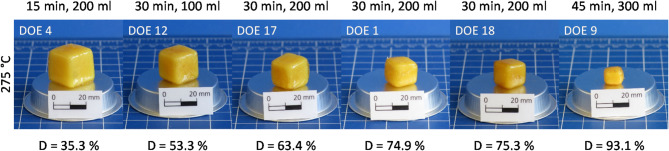
Epoxy specimens after hydrolysis at 275 °C with different decomposition durations and water volumes.

At a temperature of 275 °C (Fig. 4), decomposition proceeds depending on duration. After 15 min, about one third of the epoxy cube decomposes. After 30 min, up to 75% of the material dissolves, after 45 min over 90%. Therefore, a slightly longer treatment at 275 °C would most likely already lead to complete decomposition.

As the decomposition progresses, the specimens retain both their color and cube shape. The edge lengths decrease evenly, so that the cubes continue to shrink. The decomposition is thus still limited to the surfaces of the specimens and follows the mechanism of the heterogeneous surface degradation.

**Fig. 5:**
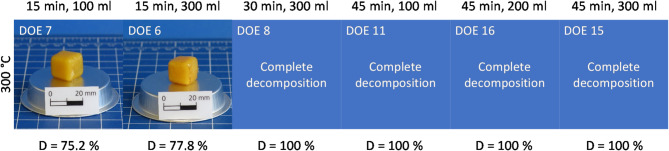
Epoxy specimens after hydrolysis at 300 °C with different decomposition durations and water volumes.

At a temperature of 300 °C (Fig. 5), the epoxy specimens decompose completely, provided the duration is 30–45 min. After a 15 min treatment, a degree of decomposition of about 75% is reached (DOE 7, 6). This corresponds quite well to the results of DOE 1 and DOE 18 after 30 min at 275 °C in the central point of the DoE.

### Glass transition temperatures

**Fig. 6:**
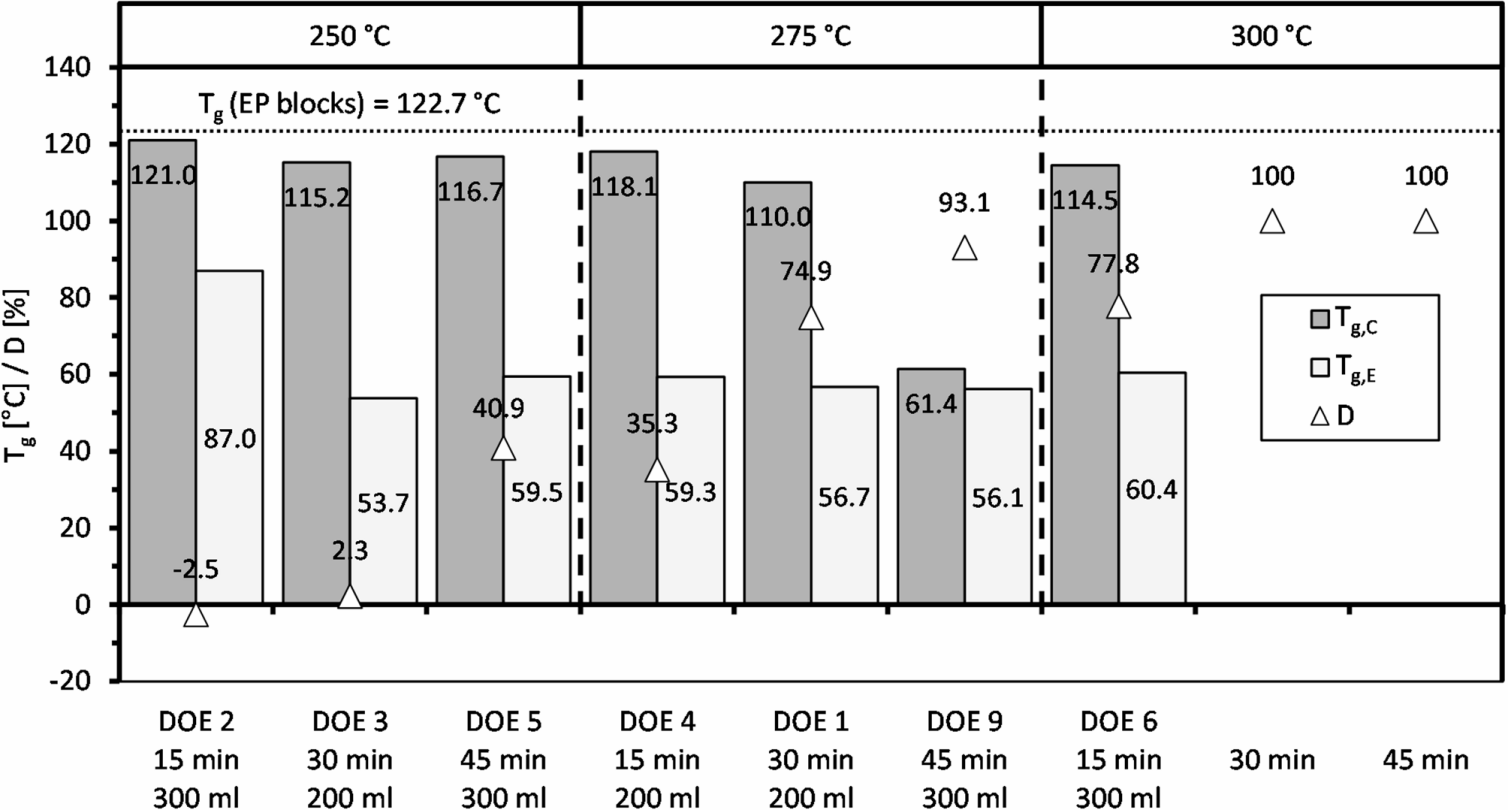
Glass transition temperatures of selected specimens in the center and at the edges of the epoxy resin cubes together with decomposition degrees.

Epoxy specimens with a decomposition duration of 15, 30 and 45 min were selected at each temperature level – if possible – and analyzed by DSC. The mean values of the glass transition temperatures from the edge (T_g, E_) and the center (T_g, C_) of these specimens after subcritical hydrolysis are displayed in Fig. 6 compared to the average T_g_ of the non-treated epoxy resin blocks.

At a temperature of 250 °C, T_g, C_ decreases only by a few degrees. Compared to the initial state of the EP blocks (122.7 °C), the value reduces by only 1.7 to 7.5 °C. On the one hand, this is due to the low hydrophilicity of the EP system. The cubes absorb only small amounts of water (2.5% maximum). Consequently, only small amounts of water can reach the center of the specimens. On the other hand, ester hydrolysis competes with water diffusion^24^. In the chemical reaction (Fig. 1), water is combined with ester groups to form carboxylic acid as well as alcohol and can therefore no longer diffuse into the center of the cubes. The decomposition characteristics described above clearly indicate that ester hydrolysis was faster than diffusion supporting this explanation. The center of the epoxy cubes may be in the first phase of the in-water degradation reported by Capiel et al.^41^. This step is mainly characterized by diffusion. They ascribed the small decrease in T_g_ of ANH-EP during this period to network plasticization due to low amounts of water absorption.

T_g, E_ after 250 °C experiments, however, decreases significantly by about 36 to 69 °C. After 15 min, a value of 87.0 °C is determined, which corresponds to about 70% of the EP blocks. After 30 and 45 min, the values of 53.7 and 59.5 °C are already below 50% of the initial state. The edges of the epoxy cube from DOE 2 are apparently in the second and third phase of the in-water degradation^41^. The polymer network is negatively affected by hydrolysis, but no mass loss is observed yet. The edges of the epoxy cubes from DOE 3 and DOE 5 already seem to have progressed to the fourth phase^41^. Lixiviation of degradation products causes a decrease in mass.

At a temperature of 275 °C, T_g, C_ hardly decreases after 15 min and only slightly after 30 min despite the high degree of decomposition of 74.9%. The center of the epoxy cubes may therefore be still in the first phase of the in-water degradation^41^. Only after 45 min the value drops to 61.4 °C, below 50% of the original T_g_ of 122.7 °C. However, this is also due to the substantially shrunk specimen size (cf. Fig. 4, DOE 9), which significantly reduces the diffusion path for the water into the center of the cube. The corresponding specimen has a remaining edge length of only about 11 mm.

T_g, E_ after 275 °C experiments apparently remains within a relatively narrow range irrespective of the decomposition duration. Values between about 56 and 59 °C are obtained, corresponding to the level after 30 and 45 min at 250 °C. This range seems to represent a kind of lower limit before the dissolution of the material. It corresponds to the fourth phase of the in-water degradation^41^.

At a temperature of 300 °C, the results of the two lower temperature levels are fundamentally confirmed. T_g, E_ agrees well with the previous values at 275 and 250 °C after 30 and 45 min – the lower limit before dissolution of the material. T_g, C_ of 114.5 °C is slightly lower than the comparable values after 15 min treatment at 275 and 250 °C. The higher temperature provides only an insignificantly higher influence of water on the center of the specimen.

These T_g_ results underline the heterogeneous surface degradation of the epoxy resin. It can be demonstrated that the water hardly affects the center of the epoxy cubes up to a very high degree of decomposition and a corresponding small specimen size. The polymer bonds clearly degrade faster due to ester hydrolysis than water diffusion proceeds.

### Decomposition kinetics

**Fig. 7:**
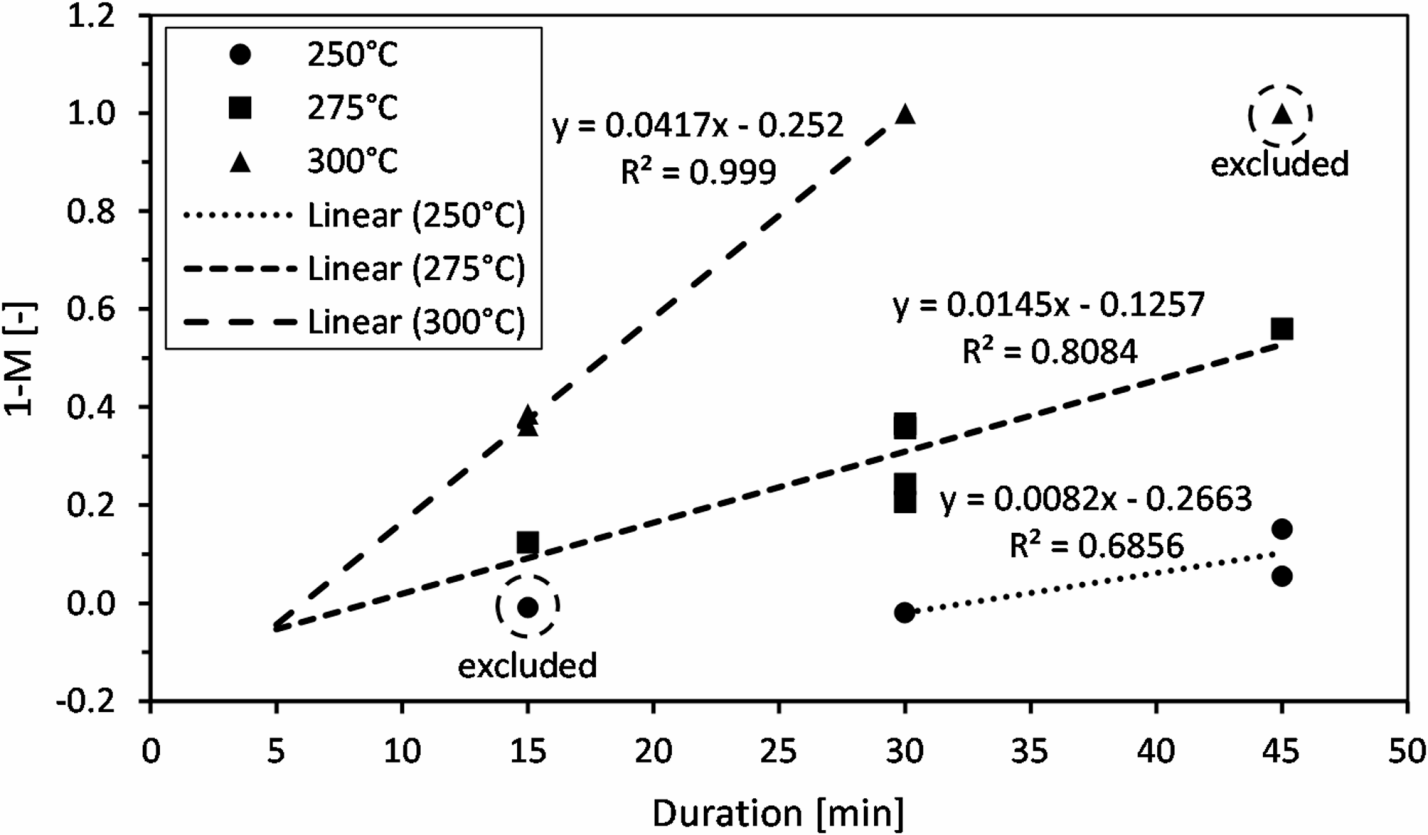
Kinetic analysis of hydrolytic decomposition reaction of epoxy resin at 250, 275 and 300 °C.

Due to the obvious surface degradation of the ANH-EP, the shrinking core model (SCM) can be applied. For evaluation of the apparent rate constant k_s_’ and the induction period t_i_ (Fig. 7), the three experiments at 300 °C with a duration of 45 min (DOE 11, 15 and 16) are not taken into account because complete decomposition was already achieved after 30 min at this temperature (DOE 8). The three experiments at 250 °C with a duration of 15 min (DOE 14, 10 and 2) are also excluded because gravimetric decomposition started only at 30 min at this temperature (DOE 3). The apparent rate constant was determined from the slope of the line of best fit, while the induction period was obtained from the intercept with the x-axis.

The calculated values are listed in Table 3. The induction time is reduced from 32.5 min at 250 °C to 8.7 and 6.0 min at 275 and 300 °C, respectively, meaning that it is decreased to a quarter and less than a fifth. The apparent rate constant nearly doubles from 0.0082 to 0.0145 min^−1^ when increasing the temperature from 250 to 275 °C. A further increase to 300 °C is associated with a further tripling of the constant to 0.0417 min^−1^. In addition to the generally higher reactivity of water at higher temperatures, a self-catalyzing effect must also be considered here. The decomposition by ester hydrolysis produces carboxylic acid (cf. Figure 1) which in turn acts as catalyzer for ester hydrolysis. Therefore, the pH value of the liquid phase decreases with starting and increasing mass loss, as observed e.g. by Capiel et al.^41^.

**Table 3:** Induction periods and apparent rate constants for hydrolytic decomposition of epoxy resin at 250, 275 and 300 °C.

Reaction temperature [°C]	Induction period t_i_ [min]	Apparent rate constant k_s_’ [min^−1^]
250	32.5	0.0082
275	8.7	0.0145
300	6.0	0.0417

**Fig. 8:**
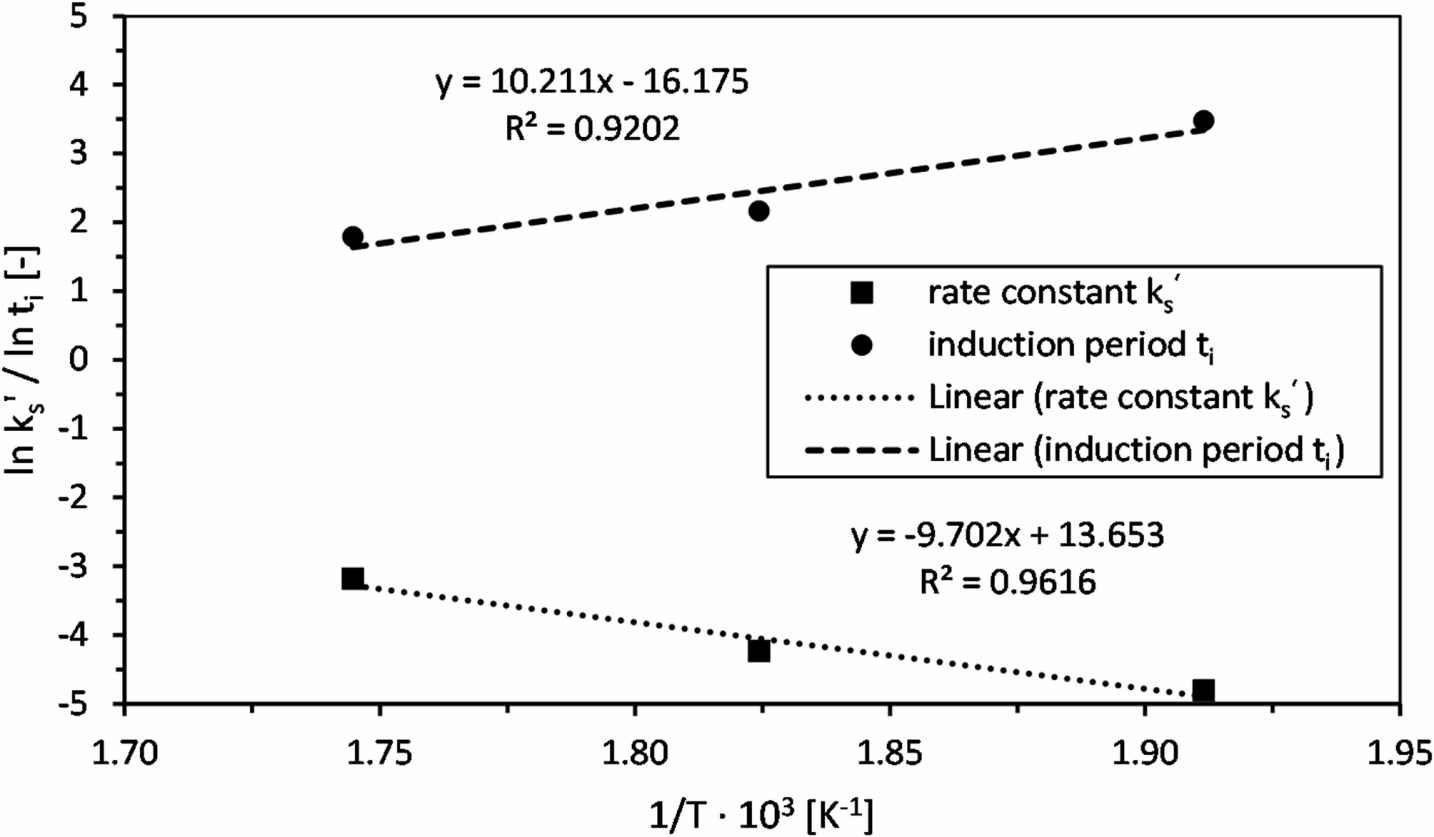
Effect of reaction temperature T on apparent rate constant k_s_’ and induction period t_i_ for hydrolytic decomposition of epoxy resin.

The dependence of the apparent rate constant and the induction period on the temperature is shown in Fig. 8. There is a linear relationship between the logarithms of k_s_’ and t_i_ with the inverse of the absolute reaction temperature. The following equations are obtained:

7 $$\:\text{ln}{k}_{s}^{{\prime\:}}=\:-\:\frac{9.702}{T}+13.653$$

8 $$\:\text{ln}{t}_{i}\:=\:\:\:\:\:\frac{10.211}{T}-16.175$$

From the straight line given by the k_s_’ data, the activation energy E_A_ and the frequency factor k_0_ for the decomposition of the epoxy resin in subcritical water can be determined from the gradient of the trend line and the intercept with the y-axis as 80.6 kJ/mol and 8.5·10^5^ min^−1^, respectively.

Compared to the use of supercritical acetone for decomposition of neat EP (118.2 kJ/mol)^34^ and the use of a supercritical acetone/water solvent mixture for recycling of CFRP (164.3 kJ/mol)^31^ E_A_ is about 30 and 50% lower, respectively. This difference can likely be attributed to the different solvents used as well as the EP resin that was cured with an amine hardener in both cases. Liu Jie et al.^33^, however, determined also a higher value of 96.3 kJ/mol for decomposition of ANH-EP by supercritical methanol and potassium hydroxide. This might indicate that the hydrolytic decomposition of this kind of EP is easier due to ester hydrolysis.

### Chemical characterization of resin decomposition products dissolved in the aqueous phase with GC-MS

**Fig. 9:**
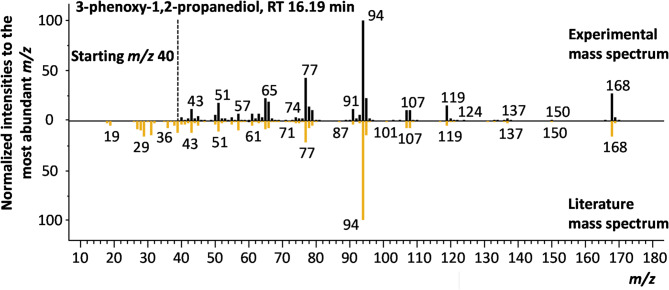
TOP – Mass spectrum of 1,2-propanediol, 3-phenoxy- (PDP) measure at retention time 16.19 min; DOWN – library mass spectra of PDP with match factor of 90.5%.

The hydrolysis process from an anhydride-cured resin is expected to produce large amounts of hydrolysis products (alcohols, carboxylic acids) from the decomposition of the polymer as shown in Fig. 1. Small compounds with functional groups have a higher solubility in water and have to be considered during treatment of the process water. Nevertheless, large decomposition products were observed as not water-soluble residues in the solvolysis process and are heavily discussed in the literature^20^. In general, 58 compounds were detected across all temperature treatments of the DoE; these are summarized in the Supplementary Tables S1, S2 and S3 online. The isomers listed in the table correspond to the most probable isomer, but deviations cannot be ruled out as no pure substances were measured for comparison. Verification using an analytical standard is planned for follow-up studies for unambiguous identification. The assignment is based on the fragmentation pattern resulting from the hard electron impact ionization and the comparison with the NIST database. Thus, a high confidence on the chemical functionalities is given. 36 compounds could be identified with more than 80% assurance (similarity scoring of the experimental with library fragment pattern). These belong to the chemical structure of ketones, alcohols, aldehydes, aromatics and phenols. As expected, only one nitrogen-containing compound (N, N-dimethylbenzylamine) could be detected in one sample, rising from impurities in the process chain and not from the polymer matrix. The higher number of compounds that are not assigned results not from uncertainties in the GC-separation or fragmentation but rather from the lack of bisphenol A based library mass spectra. This analytical lack will likely be overcome in the future using dedicated NIST library approaches currently discussed in literature^42^. The compound with the highest intensities is 1,2-propanediol, 3-phenoxy- with up to 59% of the total intensities (Fig. 9). Gong et al.^20^ reported the decomposition products dissolved in acetone; they observed similar compounds with different intensity ratios and a shift to pure hydrocarbons with more abundance. However, this deviation might be easily explained based on the specific extraction and sample preparation protocol as well as solvolysis conditions.

**Fig. 10:**
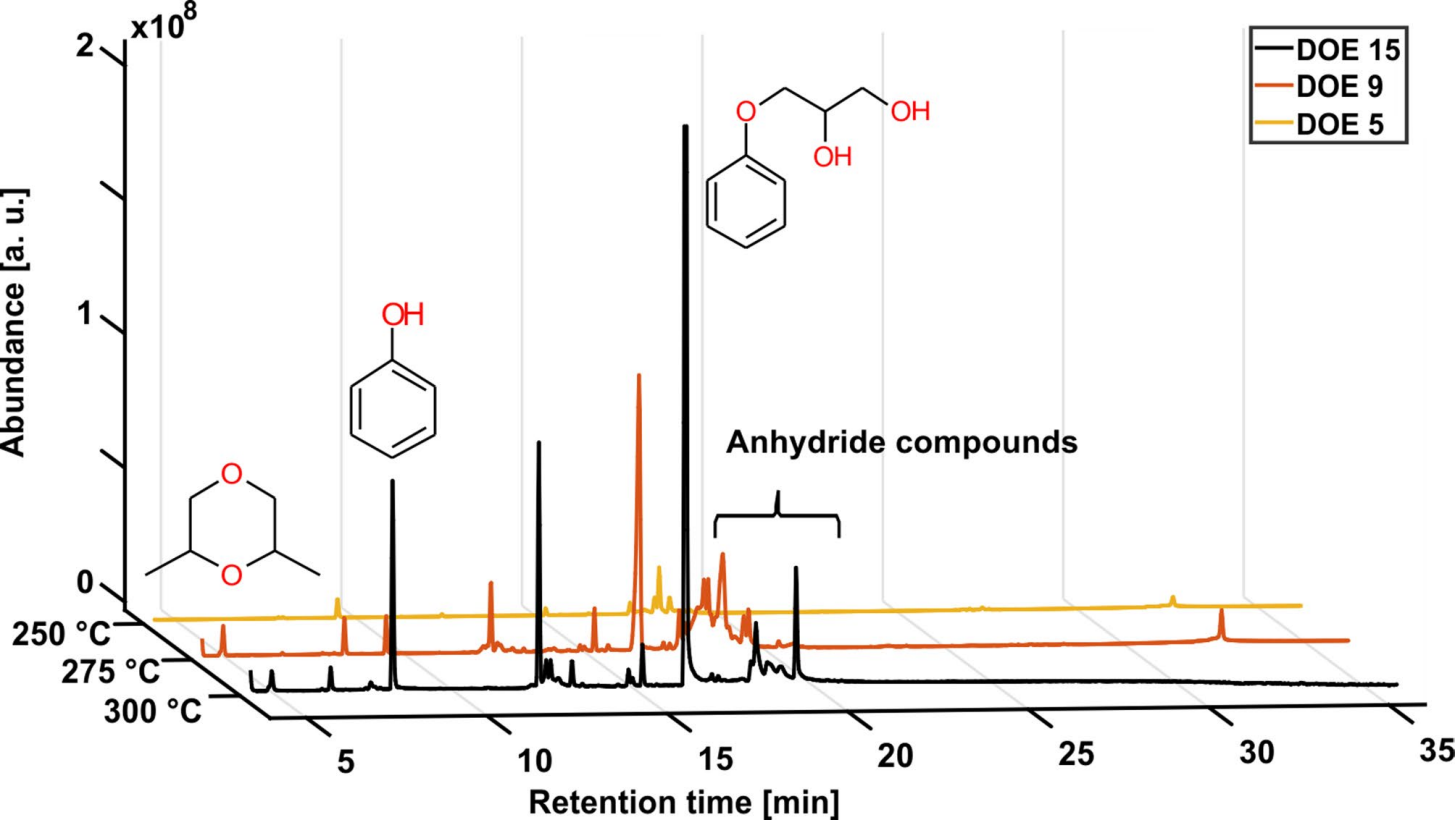
Total ion current (TIC) chromatogram with assigned compounds of the DOE’s at different temperatures (250 °C, 275 °C, 300 °C), 45 min reaction time and 300 ml water for the solvolysis process.

Figure 10 shows the total ion chromatogram (TIC) of three different reaction temperatures with identical reaction times and reaction volumes. The TIC is the corresponding mass spectral response summed over all mass channels and is used to quickly visualize the unique features of a sample. The signal intensity is directly dependent on the compound concentration, and an increase in decomposition products is observed with increasing temperature. This indicates a higher rate of chemical reactions and a more complex mixture of compounds. Hereby, the number of compounds, concentration and size change with rising temperatures, leading to a larger shift in the analysis and the complete decomposition of the test material. The trend was also observed with different reaction times and volumes shown in Supplementary Fig. S1 and Fig. S2 online. With increased time and decreased volume, an uprise in decomposition products was observed. Nevertheless, the degradation pathways were not affected by the changed condition as the same compounds were observed with similar concentrations.

A precise quantitative analysis for phenol was conducted using an external calibration method to estimate the amount of dissolved organic compounds (cf. Table 2). The findings revealed the highest concentrations, ranging from 5 to 8 g/l, for DOE 11, 15, and 16, all with a 45 min duration and a fully decomposed specimen.

## Conclusion

The analysis of the decomposition of an anhydride-cured epoxy resin by subcritical hydrolysis under variation of reaction temperature, decomposition duration and water volume showed that temperature is the most important of the three factors. The duration had a smaller but still significant effect. The water volume also contributed positively to the decomposition. However, its effect was not significant according to the statistical evaluation of the design of experiment.

The appearance of the epoxy specimens after the experiments clearly indicated a mass loss mechanism by heterogeneous surface degradation. T_g_ results demonstrated that the water hardly affected the center of the epoxy cubes up to a very high degree of decomposition and a corresponding small specimen size. The polymer bonds clearly degraded faster due to ester hydrolysis than water diffusion proceeded. Therefore, a core-shrinking model was successfully applied for analysis of the decomposition kinetics. An activation energy of 80.6 kJ/mol could be determined for the subcritical hydrolysis of the anhydride-cured epoxy resin.

The product distribution in the aqueous phase yielded a total of 58 compounds. 1,2-propanediol, 3-phenoxy- was identified as compound with the highest abundance of the 18 experiments. Higher temperatures, longer durations and lower water volumes led to an increase in decomposition products. However, as the same compounds were observed, the degradation pathways were apparently not affected by the changed conditions. The highest concentrations of phenol were found for the three 45 min experiments at 300 °C with fully decomposed specimens.

## Supplementary Information

Below is the link to the electronic supplementary material.

Supplementary Material 1

## Data Availability

The data that support the findings of this study are openly available at https://fordatis.fraunhofer.de/handle/fordatis/438.
