# Supplementary material for: Analysis of the decomposition of an anhydride-cured epoxy resin by subcritical hydrolysis
Source: Sci Rep. 2025 Jul 29;15:27647. doi: 10.1038/s41598-025-13375-8 (PMC12307930; doi:10.1038/s41598-025-13375-8)
Supplement: Supplementary file 1 — Supplementary Material 1 [file 41598_2025_13375_MOESM1_ESM.docx]

**Supplementary Material**

**Journal of Polymers and the Environment**

**Analysis of the decomposition of an anhydride-cured epoxy resin by subcritical hydrolysis**

**Simon Backens^1^* (0000-0003-2815-0919),**

**Arthur Konrad Wieland^1^ (0009-0009-4248-8095),**

**Stefan Schmidt^1^ (0009-0009-4937-5106),**

**Wilko Flügge^1^ (0009-0005-5575-3602),**

**Lukas Friederici^2,3^ (0009-0008-1221-1047),**

**Christopher Rüger^2,3^ (0000-0001-9634-9239)**

^1^Fraunhofer Institute for Large Structures in Production Engineering IGP, 18059 Rostock, Germany

^2^ Joint Mass Spectrometry Centre / Chair of Analytical Chemistry, University of Rostock, 18059 Rostock, Germany

^3^Department Life, Light & Matter (LLM), University of Rostock, 18059 Rostock, Germany

*Correspondence: simon.backens@igp.fraunhofer.de

**Table of Content**

**Table S1** Results of the extracted samples decomposed at 300 °C measured with GC MS. Compounds with less than 80% accuracy based on the fragmentation pattern were considered as not identified with the NIST library.

**Table S2** Results of the extracted samples decomposed at 275 °C measured with GC MS. Compounds with less than 80% accuracy based on the fragmentation pattern were considered as not identified with the NIST library.

**Table S3** Results of the extracted samples decomposed at 250 °C measured with GC MS. Compounds with less than 80% accuracy based on the fragmentation pattern were considered as not identified with the NIST library.

**Figure S1** Total ion current (TIC) chromatogram with assigned compounds of the DOE’s at different reaction volumes (100 ml, 200 ml, 300 ml), 45 min reaction time and 300 °C reaction temperature for the solvolysis process.

**Figure S2** Total ion current (TIC) chromatogram with assigned compounds of the DOE’s at different reaction times (15 min, 30 min, 45 min), 100 ml reaction volume and 300 °C reaction temperature for the solvolysis process.

**Table S1** Results of the extracted samples decomposed at 300 °C measured with GC MS. Compounds with less than 80% accuracy based on the fragmentation pattern were considered as not identified with the NIST library.

| **No.** | **Retention time**  **[min]** | **Compound** | **MW** | **DOE9** | **DOE1** | **DOE17** | **DOE18** | **DOE12** | **DOE4** |
| --- | --- | --- | --- | --- | --- | --- | --- | --- | --- |
| 1 | 4.19 | 1,4-Dioxane,2,5-dimethyl | 116 | - | - | - | - | - | - |
| 2 | 4.71 | 1,4-Dioxane,2,6-dimethyl | 116 | 1.26% | 1.27% | 1.22% | 1.88% | 4.11% | 1.93% |
| 3 | 5.09 | 2-Propanol, 1-(2-propenyloxy)- | 116 | 0.00% | 0.00% | 0.00% | 0.00% | 0.00% | 0.00% |
| 4 | 6.11 | Cyclohexanol | 100 | 0.08% | 0.05% | 0.00% | 0.05% | 0.00% | 0.00% |
| 5 | 6.35 | Cyclohexanol, 1-methyl- | 114 | 1.10% | 0.69% | 0.20% | 0.71% | 0.52% | 0.00% |
| 6 | 7.13 | 2-Cyclohexen-1-one | 96 | 0.00% | 0.00% | 0.01% | 0.00% | 0.00% | 0.00% |
| 7 | 7.47 | Cyclohexanol, 3-methyl- | 114 | 0.66% | 0.22% | 0.00% | 0.29% | 0.48% | 0.00% |
| 8 | 7.58 | Cyclohexanone, 3-methyl | 112 | 0.00% | 0.10% | 0.05% | 0.00% | 0.30% | 0.00% |
| 9 | 7.72 | Cyclohexanone, 4-methyl | 112 | 0.13% | 0.04% | 0.02% | 0.00% | 0.26% | 0.00% |
| 10 | 7.79 | Benzaldehyde | 106 | 0.00% | 0.00% | 0.00% | 0.00% | 0.00% | 0.00% |
| 11 | 8.10 | Phenol | 94 | 9.97% | 10.09% | 6.90% | 7.97% | 4.93% | 2.79% |
| 12 | 8.30 | 3-Methyl-3-cyclohexen-1-one | 110 | 0.00% | 0.00% | 0.00% | 0.00% | 0.00% | 0.00% |
| 13 | 8.48 | 2-Cyclohexen-1-one, 3-methyl- | 110 | 0.00% | 0.00% | 0.00% | 0.00% | 0.00% | 0.00% |
| 14 | 9.25 | Benzyl alcohol | 108 | 0.00% | 0.00% | 0.28% | 0.19% | 2.97% | 1.36% |
| 15 | 9.60 | Isomer of 2-Cyclohexen-1-one, 3-methyl- | 110 | 0.00% | 0.00% | 0.00% | 0.00% | 0.00% | 0.00% |
| 16 | 9.99 | p-Cresol | 108 | 0.00% | 0.00% | 0.00% | 0.00% | 0.00% | 0.00% |
| 17 | 10.61 | Benzofuran, 2-methyl- | 132 | 0.03% | 0.00% | 0.02% | 0.00% | 0.00% | 0.00% |
| 18 | 10.71 | 2-Methoxy-5-methylphenol | 138 | 0.06% | 0.04% | 0.03% | 0.10% | 0.12% | 0.00% |
| 19 | 11.40 | 2-Propanol, 1-[1-methyl-2-(2-propenyloxy)ethoxy]- | 174 | 0.00% | 0.00% | 0.00% | 0.00% | 0.00% | 0.00% |
| 20 | 11.62 | not identified with the NIST library (with 70 % probability for 3-Cyclohexene-1-carboxylic acid, methyl ester) | - | 0.00% | 0.00% | 0.00% | 0.00% | 0.00% | 0.00% |
| 21 | 12.06 | N,N-Diethylbenzylamine | 163 | 0.00% | 0.00% | 0.00% | 0.00% | 0.00% | 0.00% |
| 22 | 12.10 | Cyclohexene, 4-methyl- | 96 | 0.00% | 0.00% | 7.40% | 0.00% | 0.00% | 0.00% |
| 23 | 12.12 | not identified with the NIST library | - | 10.52% | 10.25% | 0.00% | 9.76% | 9.05% | 4.11% |
| 24 | 12.32 | 2-Propanone, 1-phenoxy- | 150 | 1.27% | 1.24% | 0.94% | 1.34% | 0.00% | 0.00% |
| 25 | 12.44 | p-Cumenol | 136 | 1.29% | 1.06% | 0.73% | 0.55% | 0.44% | 0.29% |
| 26 | 12.69 | not identified with the NIST library | - | 0.43% | 0.48% | 0.00% | 0.56% | 0.11% | 0.00% |
| 27 | 13.03 | Cyclohexene, 3-methyl- | 96 | 1.21% | 0.74% | 0.36% | 0.00% | 0.44% | 0.14% |
| 28 | 13.59 | p-Isopropenylphenol | - | 0.09% | 0.00% | 0.00% | 0.00% | 0.20% | 0.00% |
| 29 | 13.72 | not identified with the NIST library; typical fragments of alcohols (with 70 % probability for 1,4,7-Trimethyl-3,6-dioxaoctane-1,8-diol) | - | 0.14% | 0.00% | 0.00% | 0.00% | 0.00% | 0.00% |
| 30 | 13.87 | Benzoic acid, 4-methyl- | 138 | 0.00% | 0.00% | 0.00% | 0.00% | 0.00% | 0.65% |
| 31 | 14.32 | 3,4,5,6-Tetrahydrophthalsäure-anhydrid | 152 | 0.11% | 0.27% | 0.38% | 0.13% | 0.19% | 0.15% |
| 32 | 14.38 | not identified with the NIST library | - | 0.81% | 0.00% | 0.00% | 0.00% | 0.00% | 0.00% |
| 33 | 14.60 | 2H-1-Benzopyran-3-ol, 3,4-dihydro- | 150 | 0.50% | 0.84% | 0.50% | 0.72% | 0.35% | 0.22% |
| 34 | 14.70 | 3-Methyl-3,4,5,6-tetrahydrophthalic anhydride | 166 | 1.91% | 0.58% | 0.66% | 0.60% | 0.69% | 0.56% |
| 35 | 15.00 | Isomer to No. 34 | 166 | 0.00% | 2.68% | 3.72% | 2.50% | 4.05% | 5.09% |
| 36 | 15.21 | 3-Methyl-4-cyclohexene-1,2-dicarboxylic anhydride | 166 | 0.00% | 0.08% | 0.09% | 0.11% | 0.24% | 0.18% |
| 37 | 15.38 | 1,4-Cyclohexadiene, 1-methyl- | 94 | 0.08% | 0.13% | 0.00% | 0.16% | 0.21% | 0.48% |
| 38 | 16.19 | 1,2-Propanediol, 3-phenoxy- | 168 | 53.62% | 52.48% | 43.06% | 57.06% | 58.67% | 37.58% |
| 39 | 16.54 | Isomer to 3-Methyl-3,4,5,6-tetrahydrophthalic anhydride (No. 34) | 166 | 0.00% | 0.00% | 0.00% | 0.00% | 0.00% | 0.00% |
| 40 | 16.91 | Not identified with the NIST library – specific fragment ion is 1H-Inden-5-ol, 2,3-dihydro- | - | 1.44% | 0.49% | 0.89% | 0.59% | 0.78% | 0.91% |
| 41 | 17.09 | Phenol, 4-(2-propenyl)- | 134 | 0.74% | 0.50% | 0.71% | 0.51% | 1.45% | 0.66% |
| 42 | 17.26 | (4-tert-Butylphenoxy) acetic acid | 208 | 0.00% | 0.00% | 0.00% | 0.20% | 0.00% | 0.00% |
| 43 | 17.33 | not identified with the NIST library | - | 0.00% | 0.00% | 0.00% | 0.00% | 0.00% | 0.00% |
| 44 | 17.55 | not identified with the NIST library | - | 0.00% | 0.00% | 0.00% | 0.00% | 0.00% | 0.00% |
| 45 | 17.75 | 1,3-Isobenzofurandione, 3a,4,7,7a-tetrahydro-5-methyl- | 166 | 0.60% | 1.53% | 8.16% | 0.49% | 0.00% | 18.15% |
| 46 | 18.02 | Isomer to No. 45 | 140 | 1.26% | 0.00% | 11.85% | 0.00% | 0.00% | 0.00% |
| 47 | 18.43 | 1-Methylcyclohex-1-en-4-carboxylic acid | - | 3.08% | 8.47% | 8.66% | 5.87% | 0.00% | 16.11% |
| 48 | 18.62 | not identified with the NIST library | - | 0.00% | 0.00% | 0.00% | 0.00% | 0.00% | 3.98% |
| 49 | 18.80 | not identified with the NIST library | - | 2.00% | 0.80% | 0.00% | 2.30% | 5.85% | 1.69% |
| 50 | 19.00 | not identified with the NIST library | - | 0.00% | 0.00% | 0.00% | 0.00% | 0.00% | 1.13% |
| 51 | 19.25 | not identified with the NIST library | - | 5.55% | 4.88% | 3.15% | 5.16% | 3.58% | 1.22% |
| 52 | 20.11 | Specific phenol fragment ion; not identified with the NIST library | - | 0.06% | 0.00% | 0.00% | 0.18% | 0.00% | 0.11% |
| 53 | 20.51 | not identified with the NIST library | - | 0.00% | 0.00% | 0.00% | 0.00% | 0.00% | 0.53% |
| 54 | 21.60 | Specific phenol fragment ion; not identified with the NIST library | - | 0.00% | 0.00% | 0.00% | 0.00% | 0.00% | 0.00% |
| 55 | 23.14 | Isomer to No. 52 not identified with the NIST library | - | 0.00% | 0.00% | 0.00% | 0.00% | 0.00% | 0.00% |
| 56 | 25.5-27.5 | BPA-derivate; not identified with the NIST library |  | 0.00% | 0.00% | 0.00% | 0.00% | 0.00% | 0.00% |
| 57 | 28.00 | BPA-derivate; not identified with the NIST library | - | 0.00% | 0.00% | 0.00% | 0.00% | 0.00% | 0.00% |
| 58 | 32.40 | BPA-derivate; not identified with the NIST library | - | 0.00% | 0.00% | 0.00% | 0.00% | 0.00% | 0.00% |

**Table S2** Results of the extracted samples decomposed at 275 °C measured with GC MS. Compounds with less than 80% accuracy based on the fragmentation pattern were considered as not identified with the NIST library.

| **No.** | **Retention time**  **[min]** | **Compound** | **MW** | **DOE9** | **DOE1** | **DOE17** | **DOE18** | **DOE12** | **DOE4** |
| --- | --- | --- | --- | --- | --- | --- | --- | --- | --- |
| 1 | 4.19 | 1,4-Dioxane,2,5-dimethyl | 116 | - | - | - | - | - | - |
| 2 | 4.71 | 1,4-Dioxane,2,6-dimethyl | 116 | 2.34% | 1.85% | 0.83% | 1.47% | 0.62% | 0.43% |
| 3 | 5.09 | 2-Propanol, 1-(2-propenyloxy)- | 116 | 0.03% | 0.06% | 0.00% | 0.05% | 0.00% | 0.00% |
| 4 | 6.11 | Cyclohexanol | 100 | 0.00% | 0.00% | 0.00% | 0.00% | 0.00% | 0.00% |
| 5 | 6.35 | Cyclohexanol, 1-methyl- | 114 | 0.13% | 0.04% | 0.03% | 0.02% | 0.07% | 0.00% |
| 6 | 7.13 | 2-Cyclohexen-1-one | 96 | 0.00% | 0.00% | 0.00% | 0.00% | 0.00% | 0.00% |
| 7 | 7.47 | Cyclohexanol, 3-methyl- | 114 | 0.17% | 0.04% | 0.00% | 0.07% | 0.00% | 0.00% |
| 8 | 7.58 | Cyclohexanone, 3-methyl | 112 | 0.00% | 0.06% | 0.18% | 0.13% | 0.10% | 0.39% |
| 9 | 7.72 | Cyclohexanone, 4-methyl | 112 | 0.13% | 0.03% | 0.21% | 0.12% | 0.15% | 0.43% |
| 10 | 7.79 | Benzaldehyde | 106 | 0.00% | 0.10% | 0.10% | 0.14% | 0.00% | 0.00% |
| 11 | 8.10 | Phenol | 94 | 2.69% | 2.24% | 1.06% | 1.59% | 3.05% | 0.43% |
| 12 | 8.30 | 3-Methyl-3-cyclohexen-1-one | 110 | 0.00% | 0.03% | 0.08% | 0.08% | 0.00% | 0.13% |
| 13 | 8.48 | 2-Cyclohexen-1-one, 3-methyl- | 110 | 0.00% | 0.02% | 0.05% | 0.06% | 0.00% | 0.00% |
| 14 | 9.25 | Benzyl alcohol | 108 | 2.59% | 4.69% | 6.85% | 3.66% | 3.91% | 14.90% |
| 15 | 9.60 | Isomer of 2-Cyclohexen-1-one, 3-methyl- | 110 | 0.00% | 0.07% | 0.12% | 0.05% | 0.14% | 0.08% |
| 16 | 9.99 | p-Cresol | 108 | 0.00% | 0.05% | 0.06% | 0.07% | 0.05% | 0.05% |
| 17 | 10.61 | Benzofuran, 2-methyl- | 132 | 0.00% | 0.00% | 0.00% | 0.00% | 0.00% | 0.00% |
| 18 | 10.71 | 2-Methoxy-5-methylphenol | 138 | 0.04% | 0.00% | 0.00% | 0.00% | 0.00% | 0.00% |
| 19 | 11.40 | 2-Propanol, 1-[1-methyl-2-(2-propenyloxy)ethoxy]- | 174 | 0.00% | 0.00% | 0.04% | 0.05% | 0.00% | 0.00% |
| 20 | 11.62 | not identified with the NIST library (with 70 % probability for 3-Cyclohexene-1-carboxylic acid, methyl ester) | - | 0.06% | 0.00% | 0.00% | 0.00% | 0.00% | 0.00% |
| 21 | 12.06 | N,N-Diethylbenzylamine | 163 | 0.52% | 0.00% | 0.00% | 0.00% | 0.00% | 0.00% |
| 22 | 12.10 | Cyclohexene, 4-methyl- | 96 | 0.00% | 0.00% | 0.00% | 0.00% | 0.00% | 0.00% |
| 23 | 12.12 | not identified with the NIST library | - | 5.25% | 4.20% | 2.86% | 2.82% | 3.03% | 1.96% |
| 24 | 12.32 | 2-Propanone, 1-phenoxy- | 150 | 1.72% | 0.89% | 0.31% | 0.58% | 0.88% | 0.00% |
| 25 | 12.44 | p-Cumenol | 136 | 0.00% | 0.27% | 0.00% | 0.00% | 0.31% | 0.00% |
| 26 | 12.69 | not identified with the NIST library | - | 0.52% | 0.13% | 0.00% | 0.11% | 0.35% | 0.00% |
| 27 | 13.03 | Cyclohexene, 3-methyl- | 96 | 0.23% | 0.15% | 0.14% | 0.18% | 0.17% | 0.16% |
| 28 | 13.59 | p-Isopropenylphenol | - | 0.00% | 0.00% | 0.00% | 0.00% | 0.00% | 0.00% |
| 29 | 13.72 | not identified with the NIST library; typical fragments of alcohols (with 70 % probability for 1,4,7-Trimethyl-3,6-dioxaoctane-1,8-diol) | - | 0.00% | 0.94% | 0.55% | 0.72% | 0.00% | 0.05% |
| 30 | 13.87 | Benzoic acid, 4-methyl- | 138 | 0.00% | 1.35% | 0.00% | 0.98% | 0.16% | 0.07% |
| 31 | 14.32 | 3,4,5,6-Tetrahydrophthalsäure-anhydrid | 152 | 0.20% | 0.00% | 0.00% | 0.00% | 0.24% | 0.10% |
| 32 | 14.38 | not identified with the NIST library | - | 0.00% | 0.00% | 0.00% | 0.00% | 0.22% | 0.00% |
| 33 | 14.60 | 2H-1-Benzopyran-3-ol, 3,4-dihydro- | 150 | 0.33% | 0.28% | 0.14% | 0.17% | 0.25% | 0.00% |
| 34 | 14.70 | 3-Methyl-3,4,5,6-tetrahydrophthalic anhydride | 166 | 0.31% | 0.00% | 0.34% | 0.37% | 0.66% | 0.35% |
| 35 | 15.00 | Isomer to No. 34 | 166 | 2.86% | 5.00% | 4.20% | 3.90% | 3.83% | 8.00% |
| 36 | 15.21 | 3-Methyl-4-cyclohexene-1,2-dicarboxylic anhydride | 166 | 0.08% | 0.14% | 0.11% | 0.12% | 0.15% | 0.22% |
| 37 | 15.38 | 1,4-Cyclohexadiene, 1-methyl- | 94 | 0.46% | 0.88% | 0.59% | 0.75% | 0.87% | 0.36% |
| 38 | 16.19 | 1,2-Propanediol, 3-phenoxy- | 168 | 34.83% | 36.37% | 18.81% | 24.83% | 25.83% | 5.20% |
| 39 | 16.54 | Isomer to 3-Methyl-3,4,5,6-tetrahydrophthalic anhydride (No. 34) | 166 | 0.12% | 0.06% | 0.00% | 0.06% | 0.20% | 0.00% |
| 40 | 16.91 | Not identified with the NIST library – specific fragment ion is 1H-Inden-5-ol, 2,3-dihydro- | - | 0.47% | 0.35% | 0.00% | 0.28% | 0.31% | 0.00% |
| 41 | 17.09 | Phenol, 4-(2-propenyl)- | 134 | 0.32% | 0.26% | 0.00% | 0.19% | 0.20% | 0.00% |
| 42 | 17.26 | (4-tert-Butylphenoxy) acetic acid | 208 | 0.00% | 0.00% | 0.00% | 0.00% | 0.00% | 0.00% |
| 43 | 17.33 | not identified with the NIST library | - | 2.42% | 3.20% | 2.89% | 4.77% | 2.37% | 1.08% |
| 44 | 17.55 | not identified with the NIST library | - | 0.83% | 0.00% | 2.44% | 2.14% | 0.55% | 0.06% |
| 45 | 17.75 | 1,3-Isobenzofurandione, 3a,4,7,7a-tetrahydro-5-methyl- | 166 | 0.00% | 9.23% | 11.81% | 0.00% | 0.00% | 8.19% |
| 46 | 18.02 | Isomer to No. 45 | 140 | 12.62% | 7.64% | 6.41% | 14.04% | 15.76% | 6.78% |
| 47 | 18.43 | 1-Methylcyclohex-1-en-4-carboxylic acid | - | 16.83% | 0.91% | 15.64% | 16.18% | 0.00% | 14.16% |
| 48 | 18.62 | not identified with the NIST library | - | 0.00% | 0.00% | 1.33% | 0.00% | 24.94% | 0.00% |
| 49 | 18.80 | not identified with the NIST library | - | 1.06% | 0.00% | 2.84% | 0.74% | 0.00% | 3.42% |
| 50 | 19.00 | not identified with the NIST library | - | 0.00% | 0.00% | 0.00% | 0.82% | 0.54% | 1.64% |
| 51 | 19.25 | not identified with the NIST library | - | 2.63% | 1.77% | 0.55% | 2.35% | 4.55% | 0.00% |
| 52 | 20.11 | Specific phenol fragment ion; not identified with the NIST library | - | 0.57% | 0.86% | 0.89% | 0.63% | 0.31% | 0.59% |
| 53 | 20.51 | not identified with the NIST library | - | 1.15% | 3.57% | 3.34% | 3.03% | 0.00% | 1.43% |
| 54 | 21.60 | Specific phenol fragment ion; not identified with the NIST library | - | 0.00% | 0.00% | 0.17% | 0.11% | 0.06% | 0.00% |
| 55 | 23.14 | Isomer to No. 52 not identified with the NIST library | - | 0.42% | 1.90% | 1.79% | 1.58% | 0.00% | 0.00% |
| 56 | 25.5-27.5 | BPA-derivate; not identified with the NIST library |  | 0.99% | 3.31% | 2.65% | 2.14% | 1.39% | 5.97% |
| 57 | 28.00 | BPA-derivate; not identified with the NIST library | - | 0.23% | 0.37% | 0.11% | 0.20% | 0.15% | 0.21% |
| 58 | 32.40 | BPA-derivate; not identified with the NIST library | - | 3.85% | 6.67% | 9.50% | 7.61% | 3.60% | 23.17% |

**Table S3** Results of the extracted samples decomposed at 250 °C measured with GC MS. Compounds with less than 80% accuracy based on the fragmentation pattern were considered as not identified with the NIST library.

| **No.** | **Retention time**  **[min]** | **Compound** | **MW** | **DOE5** | **DOE13** | **DOE3** | **DOE2** | **DOE10** | **DOE14** |
| --- | --- | --- | --- | --- | --- | --- | --- | --- | --- |
| 1 | 4.19 | 1,4-Dioxane,2,5-dimethyl | 116 | - | - | - | - | - | - |
| 2 | 4.71 | 1,4-Dioxane,2,6-dimethyl | 116 | 0.51% | 0.23% | 0.73% | 0.00% | 0.00% | 0.26% |
| 3 | 5.09 | 2-Propanol, 1-(2-propenyloxy)- | 116 | 0.00% | 0.00% | 0.00% | 0.00% | 0.00% | 0.00% |
| 4 | 6.11 | Cyclohexanol | 100 | 0.00% | 0.00% | 0.00% | 0.00% | 0.00% | 0.00% |
| 5 | 6.35 | Cyclohexanol, 1-methyl- | 114 | 0.00% | 0.00% | 0.00% | 0.00% | 0.00% | 0.00% |
| 6 | 7.13 | 2-Cyclohexen-1-one | 96 | 0.00% | 0.00% | 0.00% | 0.00% | 0.00% | 0.00% |
| 7 | 7.47 | Cyclohexanol, 3-methyl- | 114 | 0.00% | 0.00% | 0.00% | 0.00% | 0.00% | 0.00% |
| 8 | 7.58 | Cyclohexanone, 3-methyl | 112 | 0.88% | 0.66% | 0.91% | 1.86% | 15.63% | 19.22% |
| 9 | 7.72 | Cyclohexanone, 4-methyl | 112 | 1.08% | 0.78% | 1.15% | 2.40% | 19.93% | 24.85% |
| 10 | 7.79 | Benzaldehyde | 106 | 0.00% | 0.00% | 0.00% | 0.00% | 0.00% | 0.00% |
| 11 | 8.10 | Phenol | 94 | 0.42% | 0.30% | 0.91% | 0.00% | 0.00% | 0.00% |
| 12 | 8.30 | 3-Methyl-3-cyclohexen-1-one | 110 | 0.35% | 0.27% | 1.22% | 0.00% | 0.00% | 1.85% |
| 13 | 8.48 | 2-Cyclohexen-1-one, 3-methyl- | 110 | 0.00% | 0.13% | 0.00% | 0.00% | 0.00% | 0.00% |
| 14 | 9.25 | Benzyl alcohol | 108 | 16.98% | 15.45% | 31.12% | 2.11% | 25.47% | 11.94% |
| 15 | 9.60 | Isomer of 2-Cyclohexen-1-one, 3-methyl- | 110 | 0.00% | 0.57% | 0.17% | 0.00% | 0.00% | 0.00% |
| 16 | 9.99 | p-Cresol | 108 | 0.00% | 0.00% | 1.61% | 0.00% | 0.00% | 0.00% |
| 17 | 10.61 | Benzofuran, 2-methyl- | 132 | 0.00% | 0.00% | 0.00% | 0.00% | 0.00% | 0.00% |
| 18 | 10.71 | 2-Methoxy-5-methylphenol | 138 | 0.00% | 0.00% | 0.00% | 0.00% | 0.00% | 0.00% |
| 19 | 11.40 | 2-Propanol, 1-[1-methyl-2-(2-propenyloxy)ethoxy]- | 174 | 0.00% | 0.00% | 0.00% | 0.00% | 0.00% | 0.00% |
| 20 | 11.62 | not identified with the NIST library (with 70 % probability for 3-Cyclohexene-1-carboxylic acid, methyl ester) | - | 0.00% | 0.00% | 0.00% | 0.00% | 0.00% | 0.00% |
| 21 | 12.06 | N,N-Diethylbenzylamine | 163 | 0.00% | 0.00% | 0.00% | 0.00% | 0.00% | 0.52% |
| 22 | 12.10 | Cyclohexene, 4-methyl- | 96 | 0.00% | 0.97% | 1.37% | 0.00% | 3.10% | 1.29% |
| 23 | 12.12 | not identified with the NIST library | - | 1.94% | 0.00% | 0.00% | 0.00% | 0.00% | 0.00% |
| 24 | 12.32 | 2-Propanone, 1-phenoxy- | 150 | 0.00% | 0.00% | 0.00% | 0.00% | 0.00% | 0.00% |
| 25 | 12.44 | p-Cumenol | 136 | 0.07% | 0.27% | 0.00% | 0.00% | 0.00% | 0.00% |
| 26 | 12.69 | not identified with the NIST library | - | 0.00% | 0.00% | 0.00% | 0.00% | 0.00% | 0.00% |
| 27 | 13.03 | Cyclohexene, 3-methyl- | 96 | 0.00% | 0.28% | 0.56% | 0.00% | 0.00% | 0.00% |
| 28 | 13.59 | p-Isopropenylphenol | - | 0.00% | 0.00% | 0.00% | 0.00% | 0.00% | 0.00% |
| 29 | 13.72 | not identified with the NIST library; typical fragments of alcohols (with 70 % probability for 1,4,7-Trimethyl-3,6-dioxaoctane-1,8-diol) | - | 0.00% | 0.00% | 0.00% | 0.00% | 0.00% | 0.00% |
| 30 | 13.87 | Benzoic acid, 4-methyl- | 138 | 0.00% | 0.00% | 0.00% | 0.00% | 0.00% | 0.00% |
| 31 | 14.32 | 3,4,5,6-Tetrahydrophthalsäure-anhydrid | 152 | 0.00% | 0.08% | 0.00% | 0.00% | 0.63% | 0.00% |
| 32 | 14.38 | not identified with the NIST library | - | 0.00% | 0.00% | 0.00% | 0.00% | 0.00% | 0.00% |
| 33 | 14.60 | 2H-1-Benzopyran-3-ol, 3,4-dihydro- | 150 | 0.00% | 0.00% | 0.00% | 0.00% | 0.00% | 0.00% |
| 34 | 14.70 | 3-Methyl-3,4,5,6-tetrahydrophthalic anhydride | 166 | 0.00% | 0.30% | 0.32% | 0.00% | 0.00% | 0.00% |
| 35 | 15.00 | Isomer to No. 34 | 166 | 0.00% | 6.88% | 9.94% | 0.00% | 31.81% | 13.35% |
| 36 | 15.21 | 3-Methyl-4-cyclohexene-1,2-dicarboxylic anhydride | 166 | 0.00% | 0.24% | 0.22% | 1.62% | 0.00% | 0.00% |
| 37 | 15.38 | 1,4-Cyclohexadiene, 1-methyl- | 94 | 0.33% | 0.99% | 0.36% | 0.00% | 0.00% | 0.00% |
| 38 | 16.19 | 1,2-Propanediol, 3-phenoxy- | 168 | 1.93% | 1.25% | 0.00% | 0.00% | 0.00% | 0.00% |
| 39 | 16.54 | Isomer to 3-Methyl-3,4,5,6-tetrahydrophthalic anhydride (No. 34) | 166 | 0.00% | 0.00% | 0.00% | 0.00% | 0.00% | 0.00% |
| 40 | 16.91 | Not identified with the NIST library – specific fragment ion is 1H-Inden-5-ol, 2,3-dihydro- | - | 0.00% | 0.00% | 0.00% | 0.00% | 1.54% | 1.01% |
| 41 | 17.09 | Phenol, 4-(2-propenyl)- | 134 | 0.00% | 0.00% | 0.00% | 0.00% | 0.00% | 0.00% |
| 42 | 17.26 | (4-tert-Butylphenoxy) acetic acid | 208 | 0.00% | 0.00% | 0.00% | 0.00% | 0.00% | 0.00% |
| 43 | 17.33 | not identified with the NIST library | - | 12.10% | 0.00% | 0.00% | 0.00% | 0.00% | 0.00% |
| 44 | 17.55 | not identified with the NIST library | - | 2.65% | 0.00% | 0.00% | 0.00% | 0.00% | 0.00% |
| 45 | 17.75 | 1,3-Isobenzofurandione, 3a,4,7,7a-tetrahydro-5-methyl- | 166 | 7.25% | 15.25% | 3.20% | 0.17% | 0.00% | 0.00% |
| 46 | 18.02 | Isomer to No. 45 | 140 | 14.87% | 2.77% | 5.84% | 87.11% | 0.00% | 0.00% |
| 47 | 18.43 | 1-Methylcyclohex-1-en-4-carboxylic acid | - | 15.75% | 26.05% | 12.98% | 0.00% | 0.00% | 0.00% |
| 48 | 18.62 | not identified with the NIST library | - | 1.21% | 0.00% | 0.00% | 0.00% | 0.00% | 0.00% |
| 49 | 18.80 | not identified with the NIST library | - | 2.39% | 0.00% | 0.00% | 0.00% | 0.00% | 0.00% |
| 50 | 19.00 | not identified with the NIST library | - | 2.93% | 5.69% | 0.00% | 0.00% | 0.00% | 0.00% |
| 51 | 19.25 | not identified with the NIST library | - | 0.00% | 0.00% | 0.00% | 0.00% | 0.00% | 0.00% |
| 52 | 20.11 | Specific phenol fragment ion; not identified with the NIST library | - | 0.00% | 0.00% | 0.00% | 0.00% | 0.00% | 0.00% |
| 53 | 20.51 | not identified with the NIST library | - | 0.00% | 0.00% | 0.00% | 0.00% | 0.00% | 0.00% |
| 54 | 21.60 | Specific phenol fragment ion; not identified with the NIST library | - | 0.00% | 0.00% | 0.00% | 0.00% | 0.00% | 0.00% |
| 55 | 23.14 | Isomer to No. 52 not identified with the NIST library | - | 0.00% | 0.00% | 0.00% | 0.00% | 0.00% | 0.00% |
| 56 | 25.5-27.5 | BPA-derivate; not identified with the NIST library |  | 6.63% | 5.35% | 14.40% | 4.73% | 0.00% | 23.27% |
| 57 | 28.00 | BPA-derivate; not identified with the NIST library | - | 0.26% | 0.18% | 0.98% | 0.00% | 1.89% | 2.46% |
| 58 | 32.40 | BPA-derivate; not identified with the NIST library | - | 9.46% | 15.05% | 12.01% | 0.00% | 0.00% | 0.00% |

**
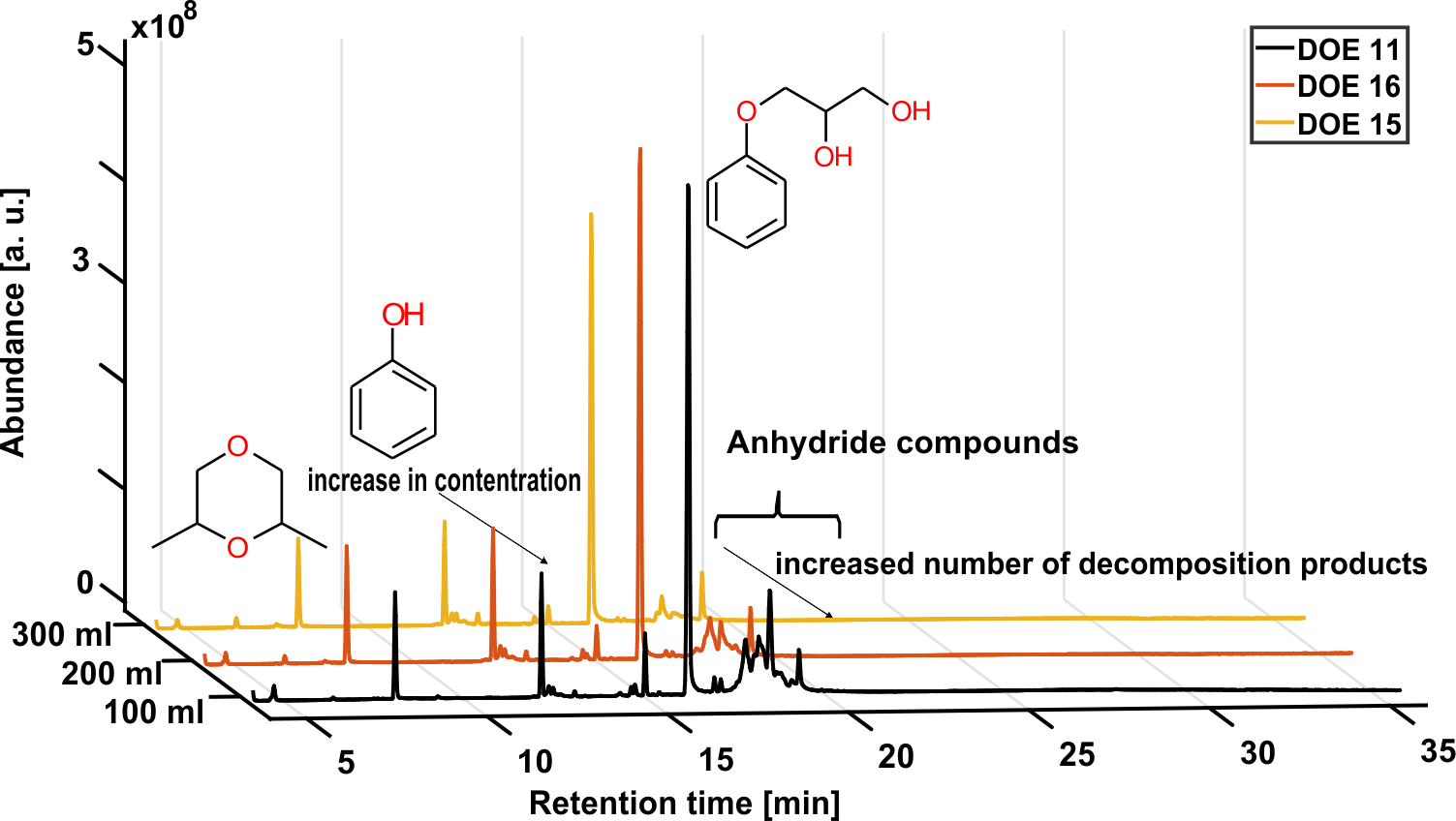
**

**Figure S1** Total ion current (TIC) chromatogram with assigned compounds of the DOE’s at different reaction volumes (100 ml, 200 ml, 300 ml), 45 min reaction time and 300 °C reaction temperature for the solvolysis process.

**
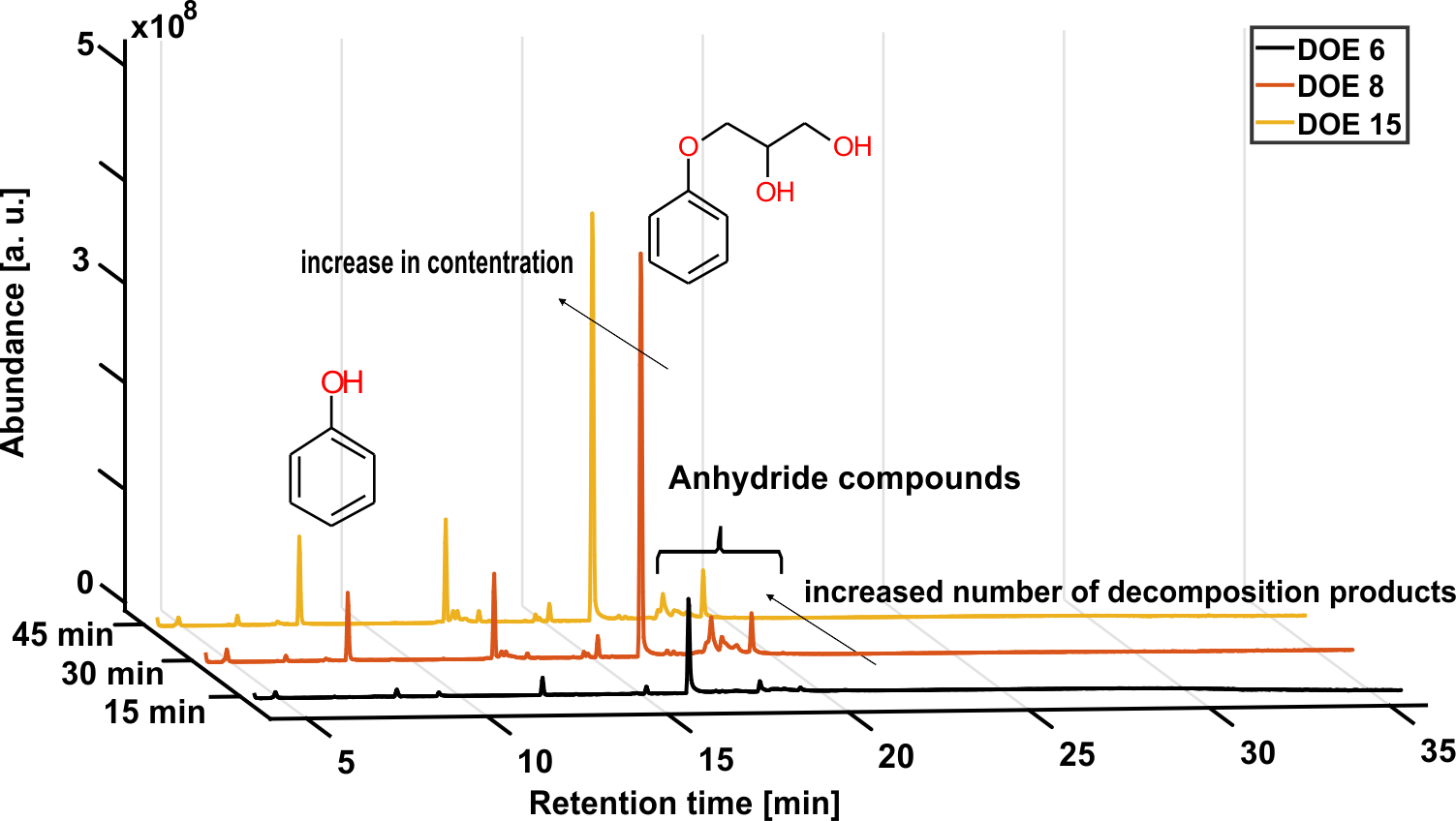
**

**Figure S2** Total ion current (TIC) chromatogram with assigned compounds of the DOE’s at different reaction times (15 min, 30 min, 45 min), 100 ml reaction volume and 300 °C reaction temperature for the solvolysis process.
